# Schizophrenia and depression, two poles of endocannabinoid system deregulation

**DOI:** 10.1038/s41398-017-0029-y

**Published:** 2017-12-18

**Authors:** María Rodríguez-Muñoz, Pilar Sánchez-Blázquez, Luis F. Callado, J. Javier Meana, Javier Garzón-Niño

**Affiliations:** 1 Neuropharmacology, Department of Translational Neurosciences, Instituto Cajal, CSIC, Madrid, E-28002 Spain; 2 Department of Pharmacology, University of the Basque Country UPV/EHU, BioCruces Health Research Institute, Barakaldo, Spain

## Abstract

The activity of certain G protein-coupled receptors (GPCRs) and of glutamate *N*-Methyl-D-aspartate receptors (NMDARs) is altered in both schizophrenia and depression. Using postmortem prefrontal cortex samples from subjects with schizophrenia or depression, we observed a series of opposite changes in the expression of signaling proteins that have been implicated in the cross-talk between GPCRs and NMDARs. Thus, the levels of HINT1 proteins and NMDAR NR1 subunits carrying the C1 cytosolic segment were increased in depressives and decreased in schizophrenics, respect to matched controls. The differences in NR1 C1 subunits were compensated for via altered expression of NR1 subunits lacking the C1 segment; thus, the total number of NR1 subunits was comparable among the three groups. GPCRs influence the function of NR1 C1-containing NMDARs via PKC/Src, and thus, the association of mu-opioid and dopamine 2 receptors with NR1 C1 subunits was augmented in depressives and decreased in schizophrenics. However, the association of cannabinoid 1 receptors (CB1Rs) with NR1 C1 remained nearly constant. Endocannabinoids, via CB1Rs, control the presence of NR1 C1 subunits in the neural membrane. Thus, an altered endocannabinoid system may contribute to the pathophysiology of schizophrenia and depression by modifying the HINT1-NR1 C1/GPCR ratio, thereby altering GPCR-NMDAR cross-regulation.

## Introduction

Neural diseases such as schizophrenia and depression coincide with alterations in the function of G protein-coupled receptors (GPCRs) and *N*-methyl-D-aspartate (NMDA)-type ionotropic glutamate receptors (NMDARs). The dopamine, gamma-aminobutyric acid, and adenosine systems, as well as metabotropic glutamate receptors, have been implicated in schizophrenia^1^. The serotonin system was initially related to depression^2^; however, later studies strongly suggested that it also participates in schizophrenia^3,4^. Serotonin directly and indirectly regulates dopaminergic neurons^5^. Thus, serotonin can participate in dopaminergic signaling dysregulation such as that observed in schizophrenic patients. GPCRs influence the strength of synaptic plasticity^6^ via regulation of NMDAR function. Thus, the increased activity of GPCRs such as dopamine 2 receptors (D2Rs) was initially proposed to decrease NMDAR activity in schizophrenia^7–9^. Similarly, in depression, the impaired function of mostly serotonergic GPCRs^10^ was thought to underlie the increases in NMDAR function^11–13^.

Nevertheless, GPCRs and NMDARs undergo bidirectional regulation wherein GPCRs that enhance NMDAR calcium influx may be negatively controlled by the recruited glutamate signaling, as has been extensively documented in the development of analgesic tolerance via mu-opioid receptors (MORs) (see e.g., ref. 14). A similar mechanism may account for the hypofunction of certain types of serotonergic receptor observed in depressive patients^2^. Thus, NMDARs appear to play a deterministic role in the onset and consolidation of these mental illnesses^15^, such as NMDAR hypofunction promotes deregulation of mostly dopamine 2 receptors (increased function) in the striatal and prefrontal regions of schizophrenic patients^3,16,17^, whereas in depressives NMDAR hyperfunction restrains primarily serotonin signaling in the prefrontal cortex^18,19^. In support of this notion, the antagonism of NMDARs by ketamine leads to rapid, robust, and relatively sustained antidepressant effects in patients with treatment-resistant major depression, in contrast to the delayed effects observed with the use of traditional antidepressants, e.g., serotonin uptake inhibitors^20,21^.

Thus, mood stability correlates with certain levels of NMDAR function, and mood instability may appear as a consequence of NMDAR hypo- or hyperfunction. Consequently, the molecular mechanisms implicated in NMDAR regulation play an essential role in determining individual behavior. NMDAR activity stimulates the release of endocannabinoids, which act through cannabinoid type 1 receptors (CB1Rs) thereby restraining NMDAR function^22^. Thus, the endocannabinoid system appears to be critical in the negative control of NMDAR function, and in the absence of CB1Rs, NMDAR-mediated excitotoxicity increases^23^.

At molecular level, a series of GPCRs functionally couple with NMDARs via NR1 subunits bearing the regulatory C1 cytosolic segment^6^, and the tandem histidine triad nucleotide-binding protein 1 (HINT1)/sigma receptor type 1 (σ1R) supports this cross-regulation^24^. Through this mechanism, cannabinoids promote CB1R coupling to NMDARs and the subsequent co-internalization of CB1Rs together with NMDAR NR1 C1 subunits^25,26^. Therefore, endocannabinoids by controlling the levels of NR1 C1 subunits may modulate the function of NMDARs. Inadequate endocannabinoid control may produce excess or insufficient dampening of NMDAR activity, thus promoting dopamine signaling, such as in schizophrenia, or diminishing serotonergic activity, as observed in depression. A growing body of evidence associates the dysregulation of the endogenous cannabinoid system with the pathogenesis of schizophrenia^27,28^, and thus, early cannabis exposure among vulnerable subjects confers an almost twofold increase in the risk of developing this illness^29^. Moreover, cannabis exposure among individuals with an established psychotic disorder can exacerbate the symptoms of schizophrenia, trigger relapse, and worsen the course of the illness^30–32^.

The possible relationship between cannabinoids and depression is also evident. Recreational cannabis is commonly believed not to precipitate signs of depression, and in fact it largely diminishes the perception of negative depressive behaviors^33,34^. A large-scale epidemiological study has found that frequent users of cannabis exhibit a less depressed mood and a more positive affect than non-consumers of cannabis^35^, and case study reports have indicated that cannabis use promotes antidepressant effects in some clinically depressed individuals^36,37^. A series of scientific reports have suggested that depression coincides with low levels of endocannabinoid activity^38,39^. In fact, the targeted deletion of *CN1R* gene induces depressive symptoms in rodents^40^, and decreased central endocannabinoid signaling has been observed in several stress-based models of depression in rodents^41,42^.

Therefore, anomalies that cause schizophrenia and depression may converge on certain molecular substrates, whose intertwined activities maintain the behavior of individuals within the limits of normality. We hypothesize that alterations in NR1 C1 subunits and in HINT1/σ1R proteins are consequences of endocannabinoid/CB1R dysfunctions, which subsequently affects cross-regulation between NMDARs and certain GPCRs. This pathway, together with other anomalies, may contribute to these mental disorders or promote such illnesses in a subset of patients affected by a dysregulated endocannabinoid system. To investigate this possibility, we performed a postmortem comparative study on prefrontal cortical samples from depressive and schizophrenic subjects. The data revealed robust and opposite changes in HINT1 and NR1 C1 protein levels in schizophrenic and depressive subjects, as well as altered associations of GPCRs, such as MOR and D2R, but not of CB1Rs, with NMDAR NR1 C1 subunits.

## Materials and methods

### Brain tissue samples

Postmortem human brain samples from the prefrontal cortex (Brodmann’s area 9) were obtained from the University of the Basque Country (UPV/EHU) brain collection, according to the national policies of research and ethical review boards for postmortem brain studies at the time the samples were obtained. A total of 72 samples were collected from 24 schizophrenic patients, 24 patients with depression, and 24 normal subjects. Sample size was estimated based on previous studies^43^. Details about clinical inclusion criteria, sample dissection, storage and toxicological screening have been described previously^43^. None of the control subjects had a history of psychiatric disorders or had received antipsychotic and/or antidepressant medication, nor did any die as a result of suicide or a neurological disorder. Before assays, the three groups were individually matched for age ( ± 5 years), sex, race, side of the brain, and postmortem interval (PMI) elapsed before obtaining the sample tissue as much as possible. Prefrontal cortices were processed to obtain the synaptosomal fraction and immunoprecipitation assays were performed as previously described^44,45^. Allocation of each sample of the triplet was blind for the investigator until statistical analysis. Demographic and toxicological information and causes of death are shown in Supplementary Table S1.

### Primary cortical cell culture

Neuron-enriched mouse cerebral cortical cultures were prepared from the brains of embryonic day-16 wild-type 129 and HINT1 knockout mice. Cerebral cortices were dissociated and seeded (1.25 × 105 cells/cm^2^) onto multiwell dishes coated with poly-D-lysine. After 3 h, the culture medium was changed to Neurobasal medium supplemented with B-27, GlutaMAX and antibiotics (100 IU/ml Penicillin and 100 µg/mL Streptomycin solution) (Invitrogen, Paisley, UK). From days 5–7 in vitro, cytosine arabinoside (5 μM) was added to the cultures to eliminate the majority of proliferating non-neuronal cells. Cultures were maintained at 37 °C in a humidified 5% CO_2_ incubator. In some cases, cells were evaluated after transfection for 72 h, with the concentrated lentiviral vector coding the HINT1 protein cDNA.

### Statistical analysis

The solubilized membranes obtained from each subject were processed individually, and the blotting data were normalized, when necessary, to the gel loading control, α-tubulin or IgGs used to immunoprecipitate the target GPCR (See Supplementary Fig. S1). Initially, the values corresponding to the schizophrenic (S), depressive (D) and control groups (C) were statistically compared by unpaired tests and the data are presented as the mean ± SEM of *N* subjects, typically 24 unless otherwise stated. This study was followed by a matched analysis, in which demographic parameters of the individuals, tissue procurement and conservation were considered to form triplets composed of one subject of each S, D, and C groups (see Supplementary Table S1 for description of triplets). For each triplet, the value of C was assigned an arbitrary value of 1, and those of matched S and D were compared to accordingly. In this case, the results are presented as the computed mean and 95% confidence interval, and the statistical paired analysis determined the confidence of the possible differences between S or D groups with respect to the C group.

In both conditions, data were analyzed by one-way ANOVA followed by post-hoc LSD. Normal distribution and similarity of variances were previously tested. Although subjects were individually matched, the potential influence of age and PMI on results was tested. Because age, but not PMI, appeared to correlate (Pearson’s r coefficient) with some of the protein expression values, ANCOVA was performed when necessary with age at the time of death as a covariate. The presence or absence of suicide was also included as variable in the ANCOVA analysis. In a post-hoc evaluation, schizophrenic subjects were differentiated between antipsychotic-free and antipsychotic-treated according to the toxicological information at death. A similar analysis was performed in depression between antidepressant-free and antidepressant-treated subjects. Statistical analyses were performed using the Sigmaplot/SigmaStat v.13 package (SPSS Science Software, Erkrath, Germany) and InVivo Stat v.3.2 (UK). Significance was defined as two sided *p* < 0.05.

The use of human and animal tissue was approved by the Ethical Committee for Research of the CSIC (SAF2012-34991 & CAM PROEX 225/14).

## Results

The study was performed on postmortem human samples of prefrontal cortices obtained from schizophrenic (S), depressive (D), and control (C) subjects. The tissues were selected so that there were no significant differences with respect to parameters such as average age, postmortem delay and storage time (Supplementary Table S1). We then evaluated a series of signaling proteins that play an essential role in synaptic communication; hence, we determined their levels in synaptosomes obtained from the prefrontal cortices of the subjects. Table 1 shows the proteins and their phosphorylations evaluated in these samples. A significant negative correlation with age was observed for CB1R and HINT1 proteins, and a positive correlation with age was obtained for MOR. The PMI did not correlate with the expression of any of proteins studied. The total levels observed for β-catenin, GSK3β, nervous tissue-specific PKCγ, neural nitric oxide synthase (nNOS), σ1R, NMDAR total NR1 levels, and GPCRs such as MOR, CB1R, serotonin 1A (5HT1AR), serotonin 2A (5HT2AR) and dopamine 2 (D2R) were reasonably consistent with these reported in previous studies performed in prefrontal cortices of schizophrenics and depressives. In our study, each GPCR was immunoprecipitated with an antibody directed to a particular sequence located in the extracellular domain, whereas blotting analysis was performed with another antibody directed against a different amino acid sequence on the receptor. We found that this procedure was more reliable than direct detection on solubilized synaptosomal membranes, particularly for low-abundance receptors, for which antibodies may provide spurious signals^45^.

**Table 1:** Expression, means and 95% confidence intervals, relative to matched controls (value of 1) of signaling proteins and GPCRs in the prefrontal cortex of schizophrenic and depressive subjects

Protein (prefrontal cortex)	Schizophrenia	Depression	References
β-catenin (total levels)	= 0.94 (0.62–1.26)	= 1.21 (0.60–1.82)	^94^ S vs C
β-catenin P-S33/S37/T41 (GSK3β)	↓ 0.76 (0.61–0.92)	= 1.34 (0.77–1.90)	
β-catenin P-S552 (Akt)	= 1.14 (0.48–1.81)	↑ 2.42 (1.19–3.66)	
β-catenin P-S675 (PKA)	↑ 2.34 (1.68–3.00)	↑ 2.58 (1.11–4.05)	
GSK3β (total levels)	= 0.92 (0.65–1.17)	= 1.15 (0.73–1.57)	^94^ S vs C
GSK3β P-S9 (Akt)	= 0.81 (0.39–1.24)	= 1.62 (0.90–2.33)	^94^ S vs C
GSK3β P-Y216	= 0.90 (0.76–1.06)	= 0.96 (0.82–1.10)	
PKCγ (total levels)	= 1.01 (0.70–1.34)	= 1.15 (0.72–1.58)	^95^ D vs C
nNOS (total levels)	= 0.99 (0.89–1.10)	= 1.01 (0.94–1.08)	^96^ S vs C
nNOS P-S1417 (Akt)	↓ 0.67 (0.45–0.89)	↑ 1.51 (1.21–1.82)	
CaMKII P-T286	= 0.98 (0.64–1.33)	↑ 1.96 (1.08–2.84)	
σ1R (total levels)	= 0.97 (0.60–1.34)	= 0.88 (0.65–1.11)	
HINT1 (total levels)	↓ 0.56 (0.49–0.63)	↑ 1.56 (1.17–1.94)	^52^ S vs C
			^54^ D vs C
NMDAR, NR1 (total levels)	= 0.95 (0.77–1.12)	= 1.08 (0.87–1.29)	^97^ S vs C
			^98^ D vs C
NMDAR, NR1 C1 (total levels)	↓ 0.59 (0.494–0.68)	↑ 1.47 (1.23–1.71)	
MOR (total levels)	= 1.07 (0.87–1.27)	= 1.24 (0.81–1.66)	^99^ S vs C
CB1R (total levels)	= 1.36 (0.70–2.04)	= 1.25 (0.77–1.75)	^100^ S vs C
			^60^ S vs C
			^61^ S & D vs C
5HT1AR (total levels)	= 1.14 (0.90–1.40)	= 1.08 (0.83–1.20)	^58^ D vs C
5HT2AR (total levels)	= 1.03 (0.91–1.16)	= 1.12 (0.96–1.28)	^58^ D vs C
			^59^ D vs C
D2R (total levels)	= 1.11 (0.94–1.29)	= 0.86 (0.64–1.07)	^100^ S vs C

Proteins were determined by western blotting. Equal loading was verified and adjusted vs. α-tubulin, or for immunoprecipitated GPCRs vs. the heavy or light chains, as required, of the biotinylated immunoglobulins targeting the GPCR, which were captured by agarose streptavidin and accompanied the SDS-PAGE procedure. Human samples were matched as described in Methods into triplets containing one depressive, one schizophrenic and one control. Within each triplet, an arbitrary value of 1 was assigned to the control value and data from the schizophrenic and the depressive subjects were then compared to the matched control. Typically, the analysis was done on 24 triplets, with the exception of 5HT1AR and 5HT2AR, which was performed on 10 triplets. The means and their 95% confidence intervals (CI) were computed for the schizophrenic and depressive values (SigmaPlot v.13/Sigmastat). Arrows indicate significant increases or decreases in protein expression vs. the control group, *p* < 0.05. The symbol = indicates that the 95% CI included the control value of 1. Previous studies that have reported similar results are shown, indicating the groups of the comparison. Additional details are given in the Methods.

**Fig. 1:**
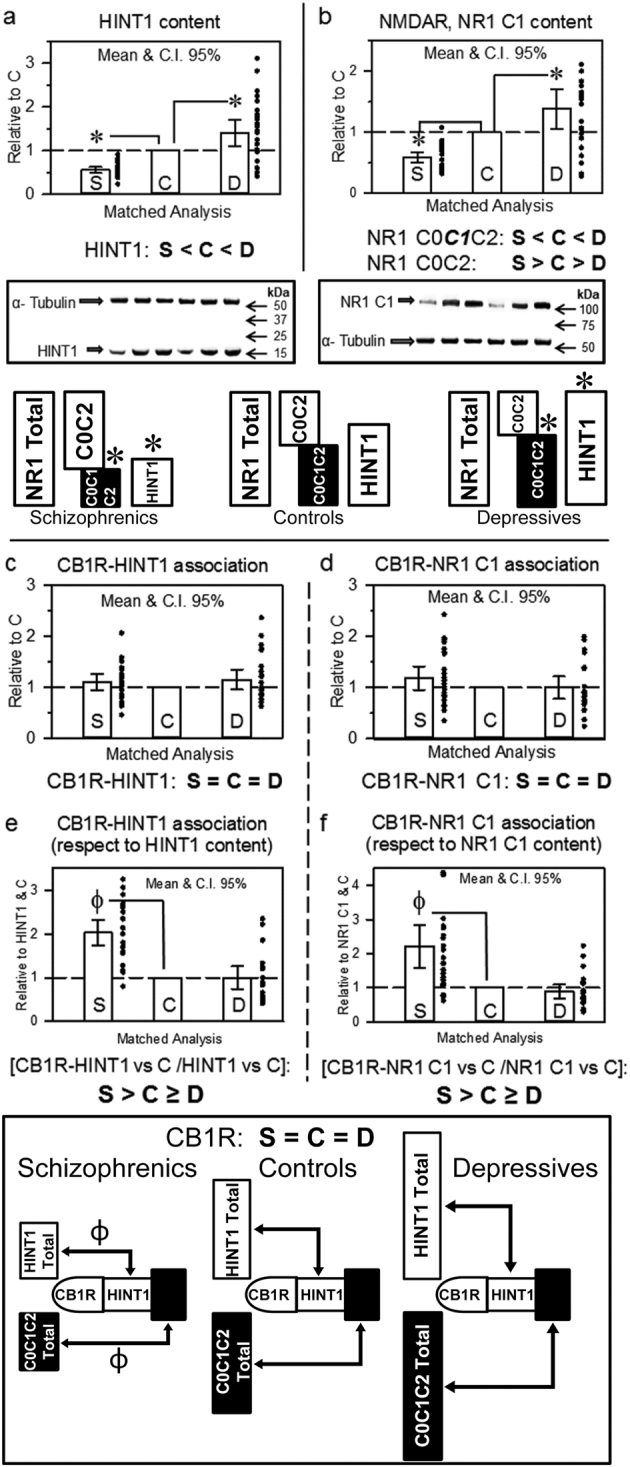
HINT1 and NMDAR NR1 C1 subunit levels and their association with CB1Rs in postmortem human prefrontal cortices of schizophrenic and depressive subjects: a comparative study vs. control individuals The analysis was first performed for each individual group, schizophrenics (S), depressives (D), and controls (C). The levels of (**a**) HINT1 and (**b**) NMDAR NR1 subunits containing C1 cytosolic segment were normalized when necessary to α-tubulin levels. Representative blots are shown. Left, S/C/D triplet 9; right S/C/D triplet 10 (see Supplementary Table S1 and Fig. S1). The assays were performed twice, and the average data for each individual were included in the subsequent matched analysis. The values of the schizophrenic and depressive subjects were compared to that of the corresponding matched control of the triplet (assigned an arbitrary value of 1), and for the S and D groups (*n* = 24) the means (bars), 95% CI (lines) and individual values (points) are shown. Analyses of covariance (ANCOVA) were performed with age, PMI and suicide as covariates. Inset: diagram indicating differences in HINT1 levels and ratios of the different NR1 subunits in the study groups. *p < 0.05 vs control in LSD post-hoc analyses. CB1R was immunoprecipitated, and CB1R-associated HINT1 (**c**) and NR1 C1 (**d**) protein levels were determined by western blotting. Data were normalized when necessary to the signals obtained probing the accompanying anti-CB1R IgGs used for immunoprecipitation with a secondary antibody (mouse anti-rabbit light chain HRP-conjugated monoclonal ab; Millipore #MAB201P). This antibody labels light chains on primary antibodies targeting the GPCR or a co-precipitated protein. Data expression and analyses as in (**a**) and (**b**). The CB1R-associated HINT1 and NR1 C1 were related to total HINT1 (**e**) and NR1 C1 (**f**) content, respectively. Inset: diagram showing the presence of CB1Rs and their association with HINT1 and NMDAR NR1 C1 subunits relative to the total content of these proteins in the study groups. ^Ф^ *p* < 0.05 vs control in LSD post-hoc analyses

The GSK3β phosphorylation site in β-catenin (S33/S37/T41) decreased by approximately 25% in schizophrenics (F[2,68] = 3.67, *p* < 0.05), whereas that of Akt (S552) increased 2.4-fold in depressives and that of PKA (S675) increased 2.5-fold in both groups of patients (F[2,68] = 4.64, *p* < 0.05). Interestingly, certain proteins or their phosphorylation levels changed in both schizophrenics and depressives but in opposite directions (Table 1). Thus, HINT1 (Fig. 1a) (F[2,67] = 12.11, *p* < 0.0001), NMDAR NR1 C1 subunits (Fig. 1b) (F[2,68] = 12.60, *p* < 0.0001) proteins and the Akt-mediated activating phosphorylation of nNOS at S1417 increased by approximately 50% in depressives, and decreased 30–50% in schizophrenics (F[2,68] = 15.96, *p* < 0.0001). The activating auto phosphorylation of CaMKII at T286 augmented almost 2-fold in depressives (F[2,68] = 8.02, *p* < 0.001). The presence of death by suicide did not modify the findings. Because NMDARs, via calcium and calmodulin, activate CaMKII and nNOS, these observations correlated with increased NMDAR activity in depressives. We and others have shown that only C1 segment-containing NR1 subunits physically interact and form stable complexes with certain GPCRs^25,44,46,47^ and that this interaction is supported by the HINT1/σ1R complex^24^.

The matched molecular analysis by ANCOVA indicated a significant decrease in HINT1 levels in the prefrontal cortices of schizophrenics (LSD post-hoc test, *p* < 0.05), whereas HINT1 levels increased in depressive subjects (LSD post-hoc test, *p* < 0.01), (Table 1 and Fig. 1a). However, the σ1R levels in the patient groups did not differ from those in the control group (Table 1). The NMDAR NR1 subunit undergoes splicing into four variants: 011/111 C0-C1-C2, 001/101 C0-C2, 010/110 C0-C1-C2’ and 000/100 C0-C2’^48^. The cytosolic C terminal region of the NR1 subunit is poorly accessible to immunoprecipitation, because it may interact with proteins such as calmodulin, CaMKII, σ1R or HINT1^49^. To circumvent this drawback, we used antibodies against an N terminal extracellular sequence common to the four spliced variants of the NR1 subunit. This particular amino acid sequence does not bind to partner proteins and is free of post translational modifications such as glycosylation or sumoylation.

The total NR1 levels were similar between the three groups of subjects (Table 1). The direct evaluation of NR1 isoforms carrying the C1 segment (for each sample the immunosignal was compared to that of the house keeper α-tubulin), revealed expression differences with schizophrenics (LSD post-hoc test, *p* < 0.01), and depressives (LSD post-hoc test, *p* < 0.05) exhibiting lower and higher levels, respectively, than controls (Fig. 1b). Because the total levels of NR1 subunits were similar among the three groups, changes in NR1 C1 subunit expression were apparently compensated for by subunits lacking the ability to interact with GPCRs. Furthermore, in the schizophrenic group, NR1 C0-C1-C2/2’ subunit levels decreased, whereas NR1 C0-C2/2’ levels increased. In contrast, in the depressive group, NR1 C0-C2/2’ decreased, favoring NMDAR NR1 C1 interactions with GPCRs (Figs. 1a, b). The presence of antipsychotic treatment in schizophrenic patients and antidepressant treatment in depressives did not alter HINT1 or NMDAR NR1 C1 expression.

Cannabinoids negatively regulate NMDAR signaling via CB1Rs; thus, we performed co-immunoprecipitation studies to evaluate the influence of the aforementioned changes on the relation of CB1Rs with NR1 C1. Our study revealed that CB1R levels were comparable among controls, schizophrenics and depressive patients (Table 1). Because σ1R rarely forms stable complexes with the aforementioned proteins in neurons, we were only able to evaluate the formation of GPCR complexes with HINT1 and NMDAR NR1 C1 subunits. Similar amounts of HINT1 and NMDAR NR1 C1 subunits co-precipitated with CB1Rs in all three groups (Figs. 1c, d). Nevertheless, the ratio of CB1Rs coupling to HINT1 (F[2,69] = 8.176, *p* < 0.0001) and NR1 C1 subunits (F[2,69] = 15.83, *p* < 0.0001) was clearly altered. Thus, since HINT1 proteins and NR1 C1 subunits were less abundant in schizophrenics, the ratio of CB1R-HINT1 (LSD post-hoc test, *p* < 0.0001) and CB1R-NR1 C1 (LSD post-hoc test, *p* < 0.0001) associations clearly exceeded that in controls. In depressive subjects, these ratios diminished, although without reaching statistical significance (Figs. 1e, f).

**Fig. 2:**
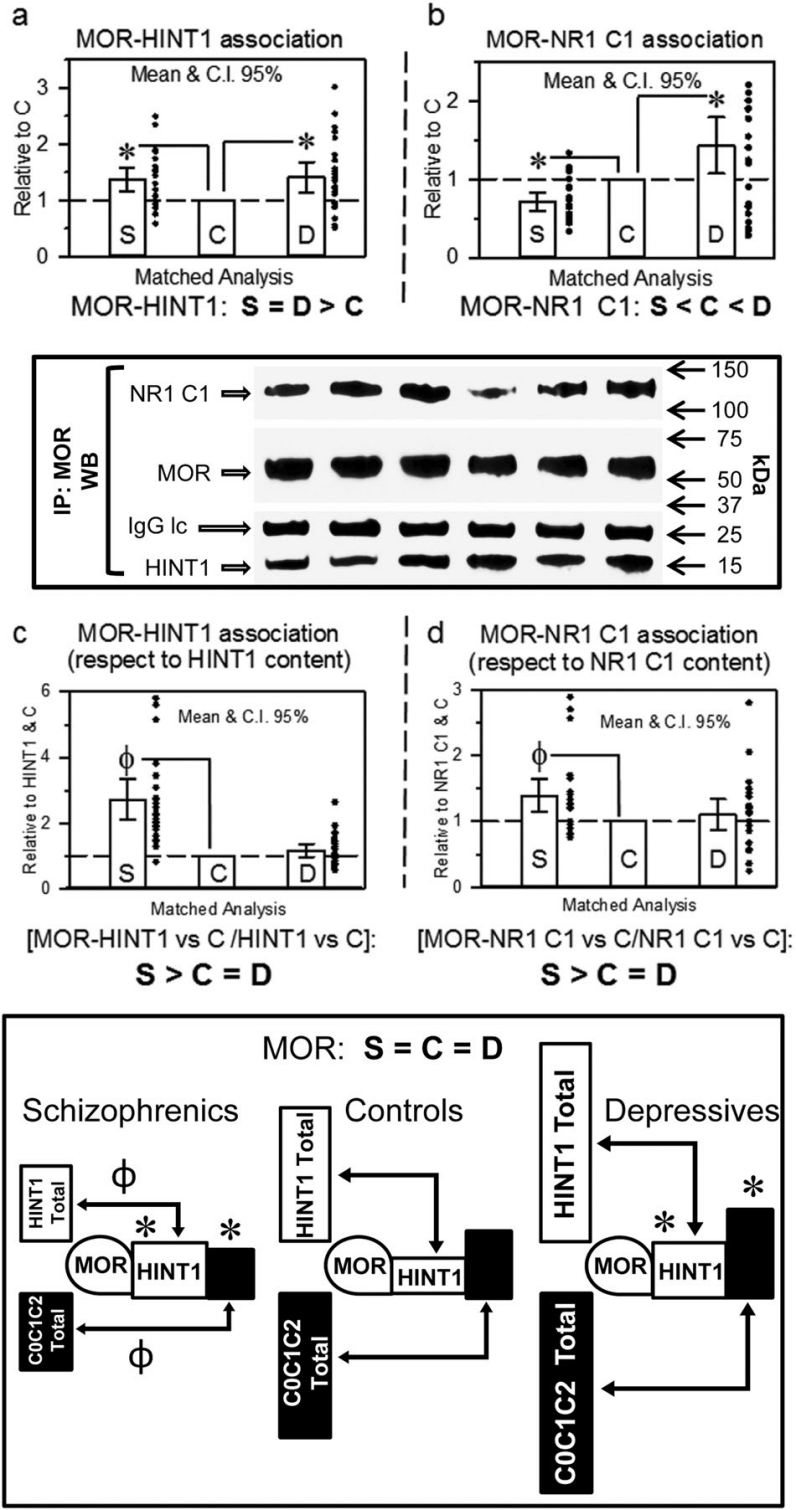
MOR association with HINT1 and NR1 C1 subunits in postmortem human prefrontal cortices of schizophrenic and depressive subjects: a comparative study vs. controls The MOR was immunoprecipitated and its association with HINT1 (**a**) and NR1 C1 subunits (**b**) was determined by western blotting. The regions of the blotting membrane incubated with different antibodies are indicated. The levels of anti-MOR IgG light chain (IgG lc) were used as a loading control. Representative blots are shown. Left, S/C/D triplet 9; right S/C/D triplet 10 (see Supplementary Table S1 and Fig. S1). Data expression and analyses as in Fig. 1. The MOR-associated HINT1 and NR1 C1 were compared to total HINT1 (**c**) and NR1 C1 (**d**) content, respectively. Inset: diagram showing the presence of MORs and their association with HINT1 and NMDAR NR1 C1 subunits relative to the total content of these proteins in the study groups. *, ^Ф^ *p* < 0.05 vs control in LSD post-hoc analyses

We then explored whether changes in NR1 C1 subunits might affect the physical coupling of GPCRs to NMDARs. The MOR was selected because it forms stable complexes with NR1 C1 subunits^14,24^ and has not been directly implicated in the mood diseases under study. Our data indicate that the MOR was evenly expressed in all three study groups (Table 1). Notably, the association of MORs with HINT1 proteins significantly increased in schizophrenic and depressive samples (F[2,69] = 6.10, *p* < 0.01) (Fig. 2a). Interestingly, schizophrenics showed less association between MORs and NMDAR NR1 C1 subunits and augmented in the depressive group (F[2,69] = 9.98, *p* < 0.001) (Fig. 2b). These associations of MORs, when normalized to HINT1 and NR1 C1 content, revealed that the proportion of MOR-coupled HINT1 was higher in schizophrenics than in controls (LSD post-hoc test, *p* < 0.0001) (Fig. 2c), and schizophrenics were found to have more NR1 C1-coupled MORs than control individuals (LSD post-hoc test, *p* < 0.05) (Fig. 2d).

**Fig. 3:**
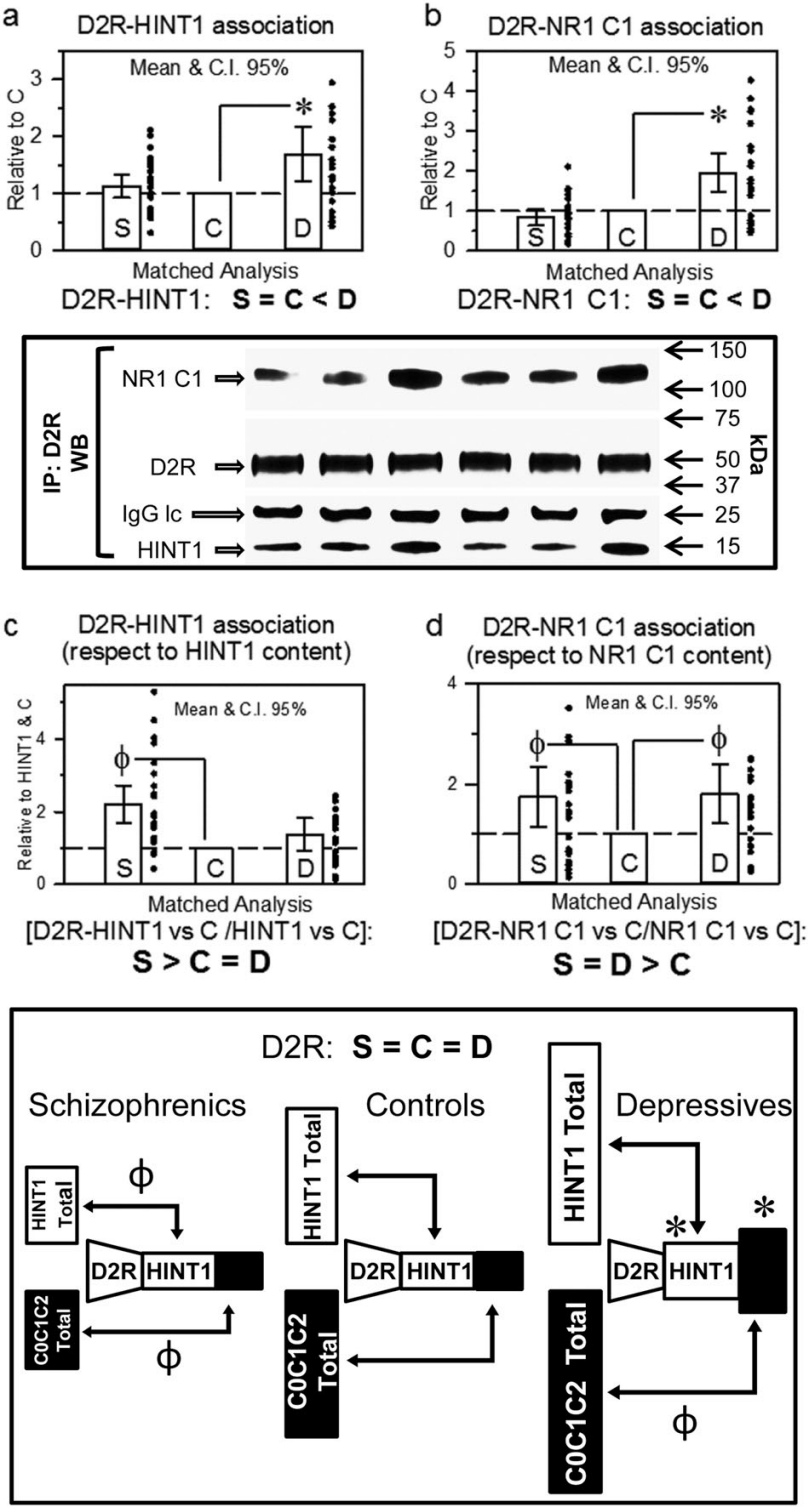
D2R association with HINT1 and NR1 C1 subunits in postmortem human prefrontal cortices of schizophrenic and depressive subjects: a comparative study vs. controls The D2R was immunoprecipitated, and its association with HINT1 (**a**) and NR1 C1 subunits (**b**) was determined by western blotting. Representative blots are shown. Left, S/C/D triplet 9; right S/C/D triplet 10 (see Supplementary Table S1 and Fig. S1). Data expression and analyses as in Figs. 1 and 2. The D2R-associated HINT1 and NR1 C1 were referred to total HINT1 (**c**) and NR1 C1 (**d**) content, respectively. Inset: diagram showing the presence of D2Rs and their association with HINT1 and NMDAR NR1 C1 subunits relative to the total content of these proteins in the study groups. *, ^Ф^ *p* < 0.05 vs control in LSD post-hoc analyses

A similar analysis was performed for the D2R receptor, and all three groups showed similar D2R levels (Table 1). In schizophrenics, the association of D2Rs with HINT1 and NR1 C1 subunits was similar to that in controls; however, in depressives, both types of D2R complexes were augmented (D2Rs with HINT1: LSD post-hoc test, *p* < 0.001; D2Rs with NR1 C1 subunits: LSD post-hoc test, *p* < 0.0001) (Figs. 3a, b). These values, when normalized to HINT1 expression (F[2,69] = 10.33, *p* < 0.0001) (Fig. 3c), showed a pattern of enhanced expression in schizophrenia similar to that seen for MORs (LSD post-hoc test, p < 0.0001). In the case of D2R-NR1 C1, normalizing the data to NR1 C1 content revealed that this association (F[2,69] = 3.62, *p* < 0.05) increases in both schizophrenics (LSD post-hoc test, *p* < 0.05) and depressives (LSD post-hoc test, *p* < 0.05) (Fig. 3d). The presence of antipsychotic treatment in schizophrenic patients and antidepressant treatment in depressives did not alter HINT1 or NMDAR NR1 C1 expression. CB1R, MOR and D2R expression and their corresponding associations to HINT1 or NR1 C1 were not modified by the presence of antipsychotic or antidepressant treatment.

**Fig. 4:**
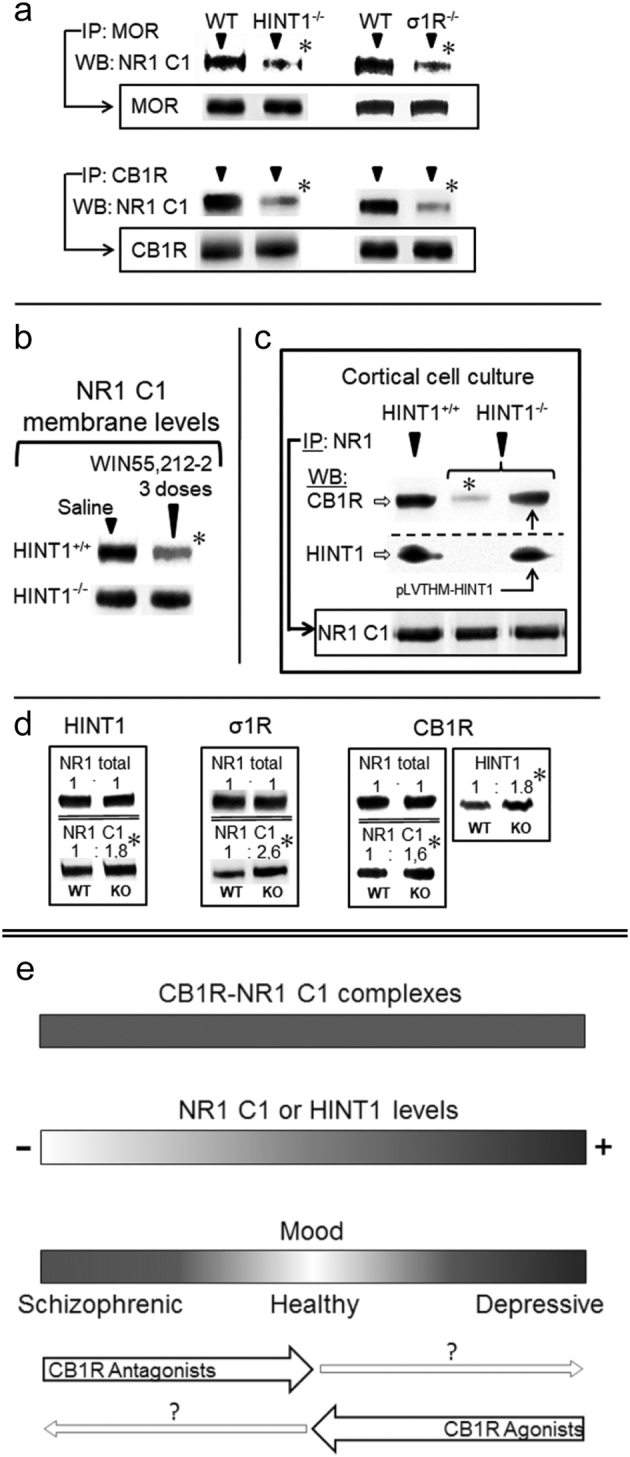
Cannabinoids, via CB1Rs negatively control the presence of NMDAR NR1 C1 subunits in neural membranes (**a**) In HINT1−/− and σ1R−/− mice, MOR/CB1R association with NR1 C1 subunits is impaired. *IP* immunoprecipitation, *WB* western blot. The immunoprecipitated levels of MORs and CB1Rs are indicated. (**b**) The cannabinoid agonist WIN55,212-2 promotes the co-internalization of CB1Rs and NR1 C1 subunits. Mice received three intracerebroventricular doses of WIN55,212-2 or saline spaced 90 min apart and were sacrificed 3 h after the last injection. Adapted from ref. ^25^. (**c**) The HINT1 protein restores the CB1R-NR1 C1 association. The HINT1 protein was introduced with lentiviral particles. Murine HINT1 was cloned in the pLVTHM vector downstream of the H1 promoter. Lentiviruses (pVLTHM-HINT1 cDNA, psPAX2, pMD2.G) were prepared in HEK-293T cells. Adapted from ref. ^23^. (**d**) Total NR1 and NR1 C1 subunits in the frontal cortices of HINT1−/−, σ1R−/− or CB1R−/− mice. For CB1R−/− mice, HINT1 levels are shown. Within each row, knockout (KO) values are compared with those of the respective wild-type (WT) mice (assigned an arbitrary value of 1). The * indicates a significant difference between the study and control group, ANOVA, followed by Dunnett’s multiple comparisons vs. control group, *p* < 0.05. Representative blots are shown. Details are given in the references indicated in Results. (**e**) Diagram showing the possible influence of HINT1/NR1 C1 levels on mental disorders such as schizophrenia and depression

Our previous studies in knockout mice confirmed the essential role of HINT1/σ1R in the cross-talk between certain GPCRs and NMDARs. In Fig. 4, we summarize the findings relevant to the role of cannabinoid system on the negative control of NMDARs. In mice, targeted deletion of the *HINT1* or *σ1R* genes impairs the association of MORs and CB1Rs with NMDAR NR1 C1 subunits^25,50^ (Fig. 4a). CB1Rs are negative regulators of NMDARs, and our previous studies showed that their activation decreases NR1 C1 subunit levels in the neural plasma membrane, but in mice lacking the HINT1 protein, the cannabinoid agonist WIN55,212-2 does not promote such a reduction in NR1 C1 subunit levels^25^ (Fig. 4b). In cortical cell cultures from wild-type and HINT1^−/−^ mice, NMDA-mediated excitotoxicity increases and cannabinoids cannot control the activity of NMDARs. In these HINT1^−/−^ cortical cells, we have shown that lentiviral expression of HINT1 restores the association between CB1Rs and NR1 C1 subunits (Fig. 4c) and rescues the neuroprotection mediated by cannabinoids^23^. In the absence of HINT1 or σ1R, the levels of NR1 C1 subunits increase, whereas total NR1 subunit levels do not change^24,51^. In mice lacking the *CNR1* gene for CB1R, the levels of HINT1 and NR1 C1 increased, whereas total NR1 subunit levels did not differ from those of wild-type mice (Fig. 4d). These observations strongly suggest a role for GPCRs, such as the CB1R, in the changes of HINT1 and NR1 C1 subunit expression observed in schizophrenic and depressive patients.

## Discussion

Our comparative study revealed that in the prefrontal cortex, the levels of a series of signaling proteins that support cross-regulation between GPCRs and NMDARs decreased in schizophrenics but increased in depressives. The σ1R/HINT1 protein complex regulates the functional coupling between these classes of receptors, and in the absence of either σ1R or HINT1, the communication between GPCRs and NMDARs is impaired or even absent^24^. In the present study we found no significant changes in σ1R levels; however, our data confirmed that HINT1 levels are lower in schizophrenics^52,53^, and higher in depressives^54^. Previous reports have primarily assessed in these mental illnesses total NR1 levels with no reference to spliced variants^55,56^. Our investigation performed on matched triplets (*n* = 24) of schizophrenics, depressives and control subjects, found no changes in total NR1 levels but found significant variations respect to controls in isoforms containing the cytosolic GPCR-interacting C1 segment. Accordingly, in response to changing situations, NR1 C0-C1-C2/2’ and NR1 C0-C2/2’ subunits may modify their relative levels without altering total NR1 subunit levels, which probably must be maintained within certain physiological limits. NMDAR activity and NR1 C1 subunits increase in depressives and decreases in schizophrenics, and changes in Akt-mediated nNOS phosphorylation provide an indication of NMDAR function. The NMDAR supplies calcium, which binds to and activates calmodulin. In this scenario, nNOS requires activation by calcium-calmodulin to produce nitric oxide (NO). From this initial point, Akt-mediated phosphorylation at S1417 enhances NO production, and the extent of the positive regulation directly correlates with NMDAR function^57^.

Using our immunoprecipitation approach to study GPCRs in synaptosomes, we detected no significant changes in the presence of MOR, D2R, 5HT1AR, 5HT2AR or CB1R in prefrontal cortices of schizophrenics or depressives. The level of 5HT2AR in depressives remains a subject of controversy, and whereas some reports have described increases^58^, others have found no significant changes^59^. Our data indicated a slight tendency to increase, but without reaching statistical confidence (mean ± 95% CI: 1.12 (0.96 – 1.28), *n* = 10). Differences in the evaluation techniques and the presence or absence of antidepressant treatment at death could explain some of the discrepancies. With respect to CB1Rs, the use of different techniques apparently leads to dissimilar findings in the prefrontal cortices of schizophrenic subjects. Immunohistochemistry analysis has indicated reductions in CB1R-related signals^60,61^, whereas these receptors show increases in ligand binding^62,63^. It is possible that in schizophrenics, an increase in CB1R coupling to G proteins enhances agonist binding to this receptor. Available antibodies for immunochemistry are directed to intracellular domains of GPCRs. Therefore, an increase in the association of signaling proteins such as G proteins with the cytosolic regions of CB1Rs would hamper the access of the antibody used to determine the CB1R levels. Without determining receptor affinity through tissue-costly assays, the data do not necessarily reflect increases in Bmax. Because moderate decreases in CB1R mRNA have also been reported in these patients^60^, we cannot exclude the possibility that CB1R levels undergo some decreases in the prefrontal cortices of schizophrenics; however, our direct assessment of the CB1R molecule revealed no such change, probably related to experimental limitations.

The NR1 C1 subunit assists NMDARs in the formation of stable complexes with GPCRs such as the MOR, CB1R, D1R and mGlu5a (see e.g., ref. 24). As a result of these associations, and in the absence of GPCR activation, the responsiveness of the GPCR-coupled NMDARs to direct activators diminishes^23^. This regulatory process depends on the HINT1/σ1R complex, and in HINT1^−/−^ mice, GPCRs only weakly stimulate NMDAR activity^24^. GPGR activation enhances NMDAR function via non-receptor tyrosine kinases, such as Src and Fyn^64^, as well as through Ser/Thr kinases, such as PKC and PKA, and in this process, the cytosolic C1 segment of the NR1 subunit also plays an essential role^6,65^. Thus, the association of MORs and D2Rs with NMDAR NR1 C1 subunits varies, as do the levels of NR1 C1 subunits, with the exception of D2R-NR1 C1 interaction in schizophrenics, which was similar to that in control subjects. The use of typical antipsychotic dopamine antagonists or atypical antipsychotics that increase serotonin 5HT1A receptor and decrease 5HT2A receptor signaling to normalize dopamine function probably restored D2R-NR1 C1 interactions. In fact, although some of the schizophrenic subjects were antipsychotic-free at death, all of them had been or were under antipsychotic treatment. Overall, the changes in the associations of these GPCRs with NMDAR NR1 C1 subunits coincided with alterations in NMDAR activity, which increase in depression and decrease in schizophrenia. Thus, NMDAR-containing NR1 C1 subunits may largely determine the expression of the glutamate symptoms of these diseases. Consequently, alterations in the composition of NR1 subunits influence the extent of GPCR-NMDAR cross-regulation, thereby affecting the GPCR-dependent activation of NMDARs and subsequent NMDAR-mediated negative feedback on GPCR signaling^6,14^.

The extent of CB1R association with NMDAR NR C1 subunits was comparable between schizophrenics, depressives and control subjects, suggesting that CB1R-NMDAR NR1 C1 coupling must be kept within certain limits to ensure appropriate endocannabinoid control over NMDAR function. This result prompts the question of whether the mechanism that controls HINT1 and NR1 C1 levels is associated with the function of CB1Rs. The serotonin 5HT1A receptor, the dopamine D3 and D4 receptors, α1 and α2 adrenergic receptors, and probably other GPCRs as well, negatively influence NMDAR function^66–69^. Among the candidates that may regulate in humans HINT1/NR1 C1 levels, the serotonin and endocannabinoid systems are important regulators of mood and emotions and are notable for being consistently related to both schizophrenia and depression^2,3,32,70^. Notwithstanding, current literature provides most convincing evidence of endocannabinoids playing an essential role in the regulation of glutamate NMDAR function. Cannabinoids decrease the strength of NMDAR signaling by regulating signaling pathways that converge intracellularly with those triggered by this glutamate receptor^71^. Interestingly, the CB1R can establish HINT1- and σ1R-dependent interactions with NMDAR NR1 C1 subunits and consequently exert negative control on NMDAR calcium influx, zinc metabolism and excitotoxicity^23,24^.

The notion that CB1Rs localize almost exclusively to presynaptic terminals, which exhibit low presence of NMDARs, may diminish the physiological relevance of *in vitro* and *ex vivo* observations showing the HINT1-mediated CB1R physical interaction with NR1 C1 subunits. Initially, the CB1R was described primarily in axon terminals; however, most recent studies have challenged this dogma^72^. Although the CB1R is concentrated in axons (pre-synapse), immunocytochemical and ultrastructural studies have demonstrated its presence in the somatodendritic compartment (post-synapse), both at the spinal^73,74^ and supraspinal levels^75,76^, and it co-localizes with NMDARs and PSD95 proteins^25^. Most newly synthetized CB1Rs first appear in postsynaptic structures such as somata and dendrites, and from this cellular compartment they are transported through endocytosis and recycling to axons, where functional CB1R accumulates in the presynaptic membrane^77^. Presynaptic CB1R inhibits calcium entry and consequently depresses neurotransmitter release^78^, and postsynaptic CB1R inhibits calcium permeation in soma and dendrites^79^. In the somatodendritic compartment, interaction between CB1Rs with other GPCRs such as the D2R^80^ and calcium channels such as NMDARs diminishes CB1R endocytosis^26,79^. The activity of endo- and exocannabinoids on these complexes leads to the co-internalization of CB1Rs and NR1 C1 subunits^25,81^.

Thus, cannabinoids influence NMDAR subunit composition and consequently NMDAR function, which is altered in schizophrenia and depression. There is no significant association between mutations in the *CNR1* gene and the predisposition to develop schizophrenia^82^. However, single nucleotide polymorphisms (SNPs) in CB1R can increase the presence of neuroticism and susceptibility to developing a depressive episode after exposure to life stress^83^ and confer an increased risk of antidepressant resistance^84^. In the prefrontal cortices of schizophrenic or depressive subjects, no significant changes in CB1R abundance were detected, thus suggesting a role for endocannabinoids in the CB1R-mediated variations in NR1 C1 levels observed in these patients. In fact, anandamide (AEA) and palmitylethanolamide levels increase in the blood^85^ and cerebrospinal fluid^86,87^ of schizophrenics, and in major depression, the circulating levels of endocannabinoids significantly decrease^88,89^. In clinical trials for the treatment of obesity with the CB1R antagonist rimonabant, a significant proportion of individuals manifested symptoms of anxiety and depression^90,91^. Notably, deficient endocannabinoid signaling also appears to precipitate a “depressive-like” phenotype in rodents^38^. Indeed, CB1R^−/−^ mice exhibit depressive-like and anxiety-like behaviors in several behavioral paradigms^92,93^, and in our study, the HINT1 and NR1 C1 subunit levels increased in these mice.

Thus, pharmacological interventions targeting the endocannabinoid system may be of therapeutic interest. The use of CB1R antagonists in schizophrenia is under consideration, and some reports have compared their effects with those of antipsychotic drugs, but given the relationship of CB1Rs with NMDARs these drugs better alleviated negative symptoms, and displayed less ability to counteract the dopamine-related positive symptoms^32^. Assuming that deficient endocannabinoid signaling contributes to depression, the adequate improvement of this system may produce antidepressant effects. In line with this hypothesis, both the direct and the indirect activation of CB1Rs produce behavioral and biochemical responses that are consistent with the effects of conventional antidepressants^38^.

In light of the quantitative differences observed in our study, parameters that support healthy normal behavior are closer to those of depressives than to those of schizophrenics (Fig. 4e). Thus, the limits of healthy mood lie between, but not necessarily midway between, the schizophrenic and depressive poles. Stimulating the endocannabinoid system, e.g., with CB1R agonists, would ameliorate depressive symptoms, and treatment with antagonists would reduce those of schizophrenia. The possible negative consequences of such exogenous CB1R ligands might be mitigated by the physiology of normal individuals, but preexisting vulnerabilities would displace mood equilibrium to either pole. The present study offers new perspectives on the analysis and comprehension of the molecular alterations that cause these illnesses and brings to fore the relevance of the interactions between GPCRs and NMDARs under the control of endocannabinoids in maintaining what is considered a normal mood.

## Electronic supplementary material

Supplementary Methods

Supplementary Figure S1

Table S1
