# Supplementary material for: Schizophrenia and depression, two poles of endocannabinoid system deregulation
Source: Transl Psychiatry. 2017 Dec 18;7:1291. doi: 10.1038/s41398-017-0029-y (PMC5802629; doi:10.1038/s41398-017-0029-y)
Supplement: Supplementary file 1 — Supplementary Methods [file 41398_2017_29_MOESM1_ESM.docx]

**Supplementary Information:**

**Supplementary Methods**

*Immunoprecipitation and western blotting*

Cerebral cortices were collected and homogenized in 10 volumes of 25 mM Tris-HCl pH 7.4, and 0.32 M sucrose supplemented with a phosphatase inhibitor mixture (P2850; Sigma), H89 (B1427; Sigma), and a protease inhibitor cocktail (P8340; Sigma). The homogenate was centrifuged at 1000 g for 10 min to remove the nuclear fraction. The supernatant (S1) was centrifuged twice at 20,000 g for 20 min to obtain the crude synaptosomal pellet (P2). The final pellet was diluted in Tris buffer supplemented with a mixture of protease inhibitors (0.2 mM phenylmethylsulphonyl fluoride, 2 μg/ml leupeptin, and 0.5 μg/ ml aprotinin), then divided into aliquots and processed for protein determinations.

For immunoprecipitation studies and to circumvent interference with signaling proteins attached to the cytosolic regions of the GPCRs, the antibodies used were directed to amino acid sequences in their extracellular domains. The affinity purified IgGs against the extracellular domains of the MOR 2^nd^ external loop (EL) (205-216: MATTKYRQGSID; GenScript Co., Piscataway, NJ, USA), CB1R 1^st^ EL (177-188: DFHVFHRKDSPN; GenScript Co.), D2R N terminal (Nt) (19-32: SRPFNGSEGKPDRP; GenScript Co.), 5HT1AR 2^nd^ EL (174-186: GWRTPEDRSNPNE), 5HT2AR Nt (25-38: SRLYPNDFNSRDAN) and the NMDAR NR1 subunit (483-496: KFGTQERVNNSNKK; GenScript Co.) were labeled with biotin (Pierce #21217 and 21339). Pilot assays were performed to optimize the amount of IgG and sample protein needed to precipitate the desired protein in a single run. The Nonidet P-40 solubilized proteins were incubated overnight at 4ºC with biotin-conjugated primary antibodies directed against the target protein. The immunocomplexes were recovered and resolved with SDS/polyacrylamide gel electrophoresis (PAGE) in 10cm×10cm×1.5mm gel slabs (7–14% total acrylamide concentration, 2.6% bisacrylamide cross-linker concentration). The separated proteins were then transferred onto 0.2 μm polyvinylidene difluoride (PVDF) membranes (162-0176; Bio-Rad, Madrid, Spain) and probed overnight at 6ºC with the selected primary antibodies diluted in Tris-buffered saline pH 7.7 (TBS) + 0.05% Tween 20 (TTBS). Those were detected using secondary antibodies conjugated to horseradish peroxidase. The western blot images, antibody binding, were visualized by chemiluminescence (#170-5061; Bio-Rad) and recorded using a ChemiImager IS-5500 (Alpha Innotech, San Leandro, CA). Protein immunosignals, and those of α-tubulin, were measured using the area of the strongest signal of each studied group of samples (average optical density of the pixels within the object area/mm2; AlphaEase FC software), the grey values of the means were then normalized within the 8 bit/256 grey levels [(256-computed value)/computed value]. Equal loading was verified and adjusted, if necessary, versus α-tubulin or the immunoprecipitated receptor. Immunosignals relative to schizophenics and depressives are shown relative to those of control subjects that were attributed an arbitrary value of 1.

*Antibodies*

The primary antibodies to detect immunoprecipitated receptors were: anti-MOR Ct aa 387–398 (GenScript Co., Piscataway, NJ), anti-CB1R Nt aa 53-66 (GenScript Co), anti-D2R (#ab30743, Abcam), anti-5HT1AR (#ab64994, Abcam), anti-5HT2AR (#ab16028, Abcam), anti-NMDAR NR1 (#MAB1586, Merck-Millipore), anti-NMDAR NR1 C1 (#MAB5046P, Merck-Millipore). Other primary antibodies used in this study were: anti-σ1R (#42-3300, Invitrogen), anti-β-catenin (#9582, Cell Signaling Technology), anti-P-S33/37/T41 β-catenin (#9561, Cell Signaling Technology), anti-P-S552 β-catenin (#9566, Cell Signaling Technology), anti-P-S675 β-catenin (#9567, Cell Signaling Technology), anti-GSK3β (#9315, Cell Signaling), anti-P-S9 GSK3β (#9336, Cell Signaling Technology), anti-P-Y216 GSK3β (#ab75745, Abcam), anti-CaMKIIpan (#3362, Cell Signaling), anti-CaMKIIα P-T286 (#3361, Cell Signaling), anti-PKCγ (#ab4145, Abcam), anti-nNOS (sc-1025, Santa Cruz Biotechnology), anti P-S1417 nNOS (#ab5583, Abcam), anti-α-tubulin (#T9026, Sigma-Aldrich). The anti-HINT1 antibody was raised in rabbits (Immunostep, Spain) against the peptide sequence GYRMVVNEGADGGG (aa 93–106). All primary antibodies were detected using the appropriate horseradish peroxidase-conjugated secondary antibodies.

*Lentiviral vector production*

RNAs isolated from mouse brain lysates were reverse-transcribed using the SuperScript® III First-Strand Synthesis System (Invitrogen) following the manufacturer’s instructions. The cDNAs for the murine HINT1 (NM_008248) was then amplified by PCR and subsequently cloned in the pLVTHM “Tet on” inducible vector downstream of the H1 promoter. Cloned inserts were sequenced to verify the integrity of each construct. Lentiviruses were prepared by cotransfection of 10 μg of pLVTHM vector (carrying either HINT1 cDNAs or the empty plasmid), 6.5 μg of second generation packaging plasmid (psPAX2) and 3.5 μg of envelope plasmid (pMD2.G) into HEK-293T cells. Transfections were carried out with a 1:3 volumetric mix of DNA and FuGENE® 6 Transfection Reagent (Roche). Lentivirus-containing supernatants were collected 48 and 72 h after transfection, filtered through 0.22-μm-pore nitrocellulose, concentrated by ultracentrifugation, aliquoted, and stored at -80 °C until used. The titer of lentivirus was determined by a hole-by-dilution titer assay.
