# Supplementary material for: Schizophrenia and depression, two poles of endocannabinoid system deregulation
Source: Transl Psychiatry. 2017 Dec 18;7:1291. doi: 10.1038/s41398-017-0029-y (PMC5802629; doi:10.1038/s41398-017-0029-y)
Supplement: Supplementary file 2 — Supplementary Figure S1 [file 41398_2017_29_MOESM2_ESM.docx]

**Supplementary Figure S1**

**
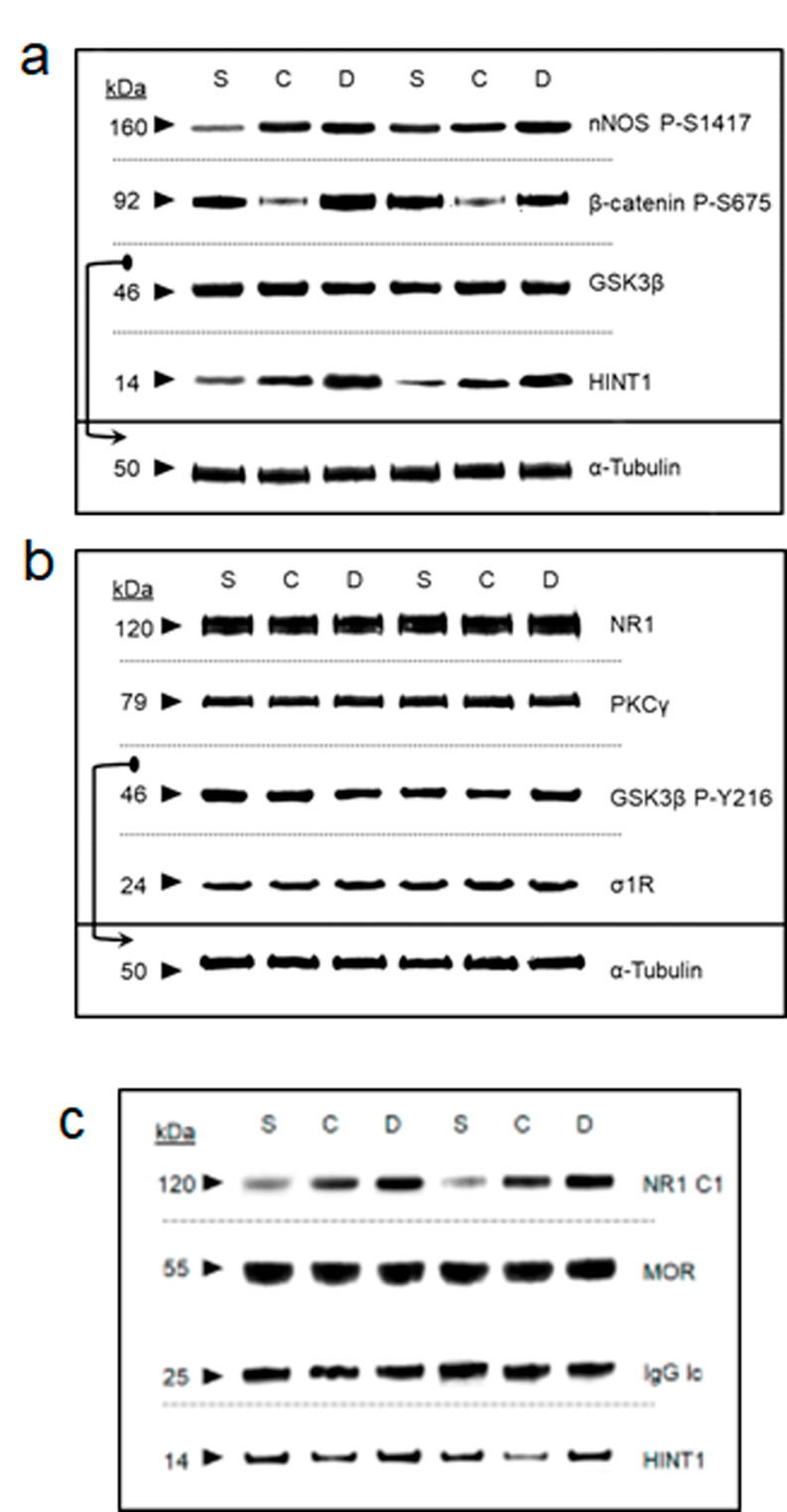
Representative blots of direct detection (a & b) and GPCR-mediated co-precipitation of proteins (c) from human prefrontal cortical synaptosomes.**

With the aim of obtaining reliable data, the blots were used only for a single determination, with the exception of α-tubulin that was obtained by reblotting the membrane. A single blot was divided into sections, e.g. upper, middle and lower, and each was incubated with a different primary antibody. Several proteins under study exhibited similar size. The blots could not be accurately cropped for their simultaneous detection; and in these cases, similar regions of a series of identical blots (generated with the same human samples) were used. In the case of direct detection, nNOS, NR1, β-catenin or their phosphorylations were studied in the upper regions. Similarly, PKCγ, GSK3β, CAMKII or their phosphorylations were analyzed in middle regions. The lower regions of these blots provided information on HINT1 and σ1R. Equal loading was verified and adjusted, if necessary, using α-tubulin as the loading control.

In the GPCR immunoprecipitation studies, the middle region was dedicated to the detection of the GPCR, the upper region for co-precipitated NR1C1 subunits and the lower to HINT1. The secondary antibodies were directed to either the heavy or light IgG chains of the primary antibodies, as needed. Thus, the secondary antibodies also reacted primarily with the separated IgG light chains of the accompanying antibodies used to immunoprecipitate de target GPCR and provided the loading control for the samples in the gel.

(a) Left, triplet 1; Right, triplet 6. (b) Left, triplet 2; Right, triplet 5. (c) Left, triplet 12; Right, triplet 14 (for details, see Supplementary Table S1).
