# Supplementary material for: Schizophrenia and depression, two poles of endocannabinoid system deregulation
Source: Transl Psychiatry. 2017 Dec 18;7:1291. doi: 10.1038/s41398-017-0029-y (PMC5802629; doi:10.1038/s41398-017-0029-y)
Supplement: Supplementary file 3 — Table S1 [file 41398_2017_29_MOESM3_ESM.docx]

**Supplementary Table S1**.

Demographic characteristics, postmortem delay (PMD), storage time, RNA integrity number (RIN), cause of death/mechanism, brain pH and toxicological analysis of the schizophrenic subjects (S), subjects with major depressive disorder (D) and matched control subjects (C).

| **Case/Control** | **Gender** | **Age** | **PMD** | **Storage time** | **RIN** | **Cause of death / Mechanism** | **Drugs in blood** |
| --- | --- | --- | --- | --- | --- | --- | --- |
| **(Sch/MDD/C)** | **(F/M)** | **(years)** | **(hours)** | **(months)** |  |  |  |
| S1  D1  C1  S2  D2  C2  S3  D3  C3  S4  D4  C4  S5  D5  C5  S6  D6  C6  S7  D7  C7  S8  D8  C8  S9  D9  C9  S10  D10  C10  S11  D11  C11  S12  D12  C12  S13  D13  C13  S14  D14  C14  S15  D15  C15  S16  D16  C16  S17  D17  C17  S18  D18  C18  S19  D19  C19  S20  D20  C20  S21  D21  C21  S22  D22  C22  S23  D23  C23  S24  D24  C24 | M  M  M  M  M  M  M  M  M  M  M  M  M  M  M  M  M  M  F  F  F  M  M  M  F  F  F  F  F  F  M  M  M  M  M  M  M  M  M  M  M  M  M  M  M  M  M  M  F  F  F  F  M  M  F  F  F  M  M  M  F  F  F  F  F  F  F  F  F  M  M  M | 44  43  44  30  30  30  31  35  32  23  21  23  42  43  42  33  35  33  28  26  28  50  47  47  30  34  32  75  74  75  44  43  43  73  73  72  48  48  47  42  43  42  33  33  33  45  48  45  74  74  73  75  73  73  38  35  35  66  65  65  56  58  58  69  68  68  48  50  50  60  60  60 | 24  34  21  18  4  11  14  20  28  16  13  17  25  15  27  14  13  4  22  26  55  3  18  63  17  6  18  48  22  39  7  15  10  17  16  16  20  32  26  25  34  27  14  14  4  3  15  30  30  22  38  48  60  49  23  23  22  57  30  49  13  27  20  11  23  16  17  19  31  7  18  51 | 192  117  116  154  150  154  150  127  151  144  122  129  136  136  187  138  165  123  130  228  237  132  129  193  166  120  145  116  119  180  155  124  154  148  127  194  146  251  201  136  118  211  136  139  123  130  118  140  94  107  132  116  230  202  191  241  166  200  216  217  143  215  200  134  207  154  132  147  205  127  132  135 | N/A  8.4  8.5  8.2  8.0  8.0  7.7  8.2  8  9.1  8.1  9.1  N/A  N/A  N/A  7.4  N/A  9.1  N/A  N/A  N/A  N/A  N/A  N/A  N/A  N/A  N/A  8.6  8  8.3  8.2  7.6  7.1  N/A  N/A  N/A  8.3  N/A  N/A  N/A  8.4  N/A  7.4  N/A  9.1  9.3  7.9  6.3  8.3  8  8.6  N/A  N/A  N/A  N/A  N/A  7.8  5.9  N/A  N/A  8.5  7.5  7.2  N/A  N/A  N/A  N/A  N/A  N/A  N/A  N/A  N/A | Suicide/Overdose  Suicide/Hanging  Accident/Traffic  Suicide/Jumping  Suicide/Jumping  Accident/Electrocution  Suicide/Jumping  Suicide/GSW  Accident/Traffic  Suicide/Jumping  Suicide/Hanging  Accident/Electrocution  Natural/CRF  Suicide/Train  Accident/Traffic  Suicide/Hanging  Suicide/Hanging  Accident/Traffic  Suicide/Jumping  Suicide/Jumping  Accident/Traffic  Suicide/Drowned  Suicide/Hanging  Accident/Traffic  Suicide/Jumping  Suicide/overdose  Accident/Traffic  Natural/CRF  Suicide/Jumping  Accident/Traffic  Natural/CRF  Suicide/Train  Accident/Traffic  Natural/CRF  Suicide/Drowned  Natural/CRF  Suicide/Train  Suicide/Hanging  Accident/Work  Natural/CRF  Suicide/Hanging  Accident/Traffic  Suicide/Hanging  Suicide/Jumping  Accident/Traffic  Suicide/Jumping  Natural/CRF  Accident/Traffic  Natural/CRF  Suicide/Jumping  Accident/electric shock  Natural/CRF  Suicide/GSW  Accident/Traffic  Suicide/Jumping  Suicide/Jumping  Accident/Traffic  Suicide/Hanging  Suicide/overdose  Accident/Traffic  Natural/CRF  Suicide/Hanging  Natural/CRF  Natural/CRF  Suicide/Jumping  Natural/Hemorrhage  Suicide/Jumping  Suicide/Jumping  Accident/Traffic  Natural/CRF  Suicide/Hanging  Accident/Sport | BZD, ETH (0.87g/l), VEN,CMI  CIT, BZD  (-)  OLZ  (-)  THC  BZD  FLU, BZD, ETH (0.74 g/l)  AMP, ETH (0.68 g/l)  SLP  (-)  (-)  LVZ  BZD, ETH (1.02 g/l)  (-)  BZD  (-)  (-)  (-)  (-)  (-)  BZD  CIT  ETH (1.16 g/l)  HLP, BZD  VEN, CIT, BZD, CLZ, TOP  ETH (1.21 g/l)  (-)  SER, TRA  (-)  LVZ, CLO, BIP, BZD  BZD  ETH (1.05 g/l)  (-)  AMI, TRA, BZD  (-)  (-)  BZD  (-)  LVZ  CIT, BZD  (-)  BZD  VEN, BZD  (-)  BZD  (-)  ETH (3.09 g/l)  (-)  SER  (-)  (-)  CIT  (-)  (-)  (-)  (-)  OLZ  CMI, TIA, BZD, ETH (2.01 g/l)  (-)  CLZ  (-)  (-)  THI, AMI, BZD  SER  (-)  OLZ, CLO  VEN, MIR  (-)  (-)  FLU, OLZ, BZD  ETH (1.02 g/l) |
| Group S | 15M/9F | 48±3 | 21±3 | 144±5 | 8.1±0.3 |  |  |
| Group D | 16M/8F | 48±3 | 22±2 | 158±10 | 8.0±0.1 |  |  |
| Group C | 16M/8F | 48±3 | 28±3 | 169±7 | 8.1±0.2 |  |  |

Group values are means±SEM. F (female), M (male), CRF (cardio-respiratory failure), GSW (gunshot wound), N/A (not available). Drugs in blood are coded as AMI (amitryptiline), AMP (amphetamine), BIP (biperiden), BZD (non-specified benzodiazepines or metabolites), CIT (citalopram), CLO (clotiapine), CLZ (clozapine), CMI (clomipramine), ETH (ethanol), FLU (fluoxetine), HLP (haloperidol), LVZ (levomepromazine), MIR (mirtazapine), OLZ (olanzapine), SER (sertraline), SLP (sulpiride), THC (tetrahidrocannabinol), THI (thioridazine), TIA (tiapride), TOP (topiramate), TRA (trazadone) and VEN (venlafaxine).
